# The validity of the SF-12 and SF-6D instruments in people living with HIV/AIDS in Kenya

**DOI:** 10.1186/s12955-017-0708-7

**Published:** 2017-07-17

**Authors:** Anik R. Patel, Richard T. Lester, Carlo A. Marra, Mia L. van der Kop, Paul Ritvo, Lidia Engel, Sarah Karanja, Larry D. Lynd

**Affiliations:** 1 Department of Medicine, University of British Columbia, 828 West 10th Avenue, Vancouver, BC V5Z 1M9 Canada; 2 Faculty of Pharmaceutical Science, University of British Columbia, 2405 Westbrook Mall, Vancouver, Canada; 3 School of Pharmacy, University of Otago, Dunedin, New Zealand; 4 Department of Public Health Sciences/Global Health (IHCAR), Karolinska Institutet, Widerströmska Huset, Tomtebodavägen 18A, 171-77 Stockholm, Sweden; 5 School of Kinesiology & Health Sciences, York University, 160 Campus Walk, North York, ON M3J 1P3 Canada; 6 Faculty of Health Sciences, Simon Fraser University, 8888 University Drive, Burnaby, BC V5A 1S6 Canada; 7 Deakin University, Geelong Australia, Faculty of Health, School of Health and Social Development, 221 Burwood Highway, Burwood, Victoria, 3125 Australia; 8 Monitoring, Evaluation and Research Unit, Amref Health Africa in Kenya, P.O. Box 30125-00100, Nairobi, Kenya; 9 Center for Health Evaluation and Outcomes Science, St. Paul’s Hospital, 588 – 1081 Burrard Street, Vancouver, BC V6Z 1Y6 Canada

**Keywords:** Quality of life, Short-form 12, Kiswahili, HIV, Health state utility, SF6D

## Abstract

**Background:**

Health-related quality of life (HRQoL) and health state utility value (HSUV) measurements are vital components of healthcare clinical and economic evaluations. Accurate measurement of HSUV and HRQoL require validated instruments. The 12-item Short-Form Health Survey (SF-12) is one of few instruments that can evaluate both HRQoL and HSUV, but its validity has not been assessed in people living with HIV/AIDS (PLWHA) in east Africa, where the burden of HIV is high.

**Methods:**

This cross-sectional study used baseline data from a randomized trial involving PLWHA in Kenya. Data included responses from a translated and adapted SF-12 survey as well as key demographic and clinical data. Construct validity of the survey was examined by testing the SF-12’s ability to distinguish between groups known in advance to have differences in their health based on their disease severity. We classified disease severity based on established definitions from the US Center for Disease Control (CDC) and WHO, as well as a previously studied viral load threshold. T-tests and ANOVA were used to test for differences in HRQoL and HSUV scores. Area under the receive operator curve (AUC) was used to test the discriminative ability of the HRQoL and HSUV instruments.

**Results:**

Differences in physical component scores met the minimum clinically important difference among participants with more advanced HIV when defined by CD4 count (4.3 units) and WHO criteria (compared to stage 1, stages 2, 3 and 4 were 2.0, 7.2 and 9.8 units lower respectively). Mental score differences met the minimum clinically important difference between WHO stage 1 and stage 4 patients (4.4). Differences in the HSUV were statistically lower in more advanced HIV by all three definitions of severity. The AUC showed poor to weak discriminatory ability in most analyses, but had fair discriminatory ability between WHO clinical stage 1 and clinical stage 4 individuals (AUC = 0.71).

**Conclusion:**

Our findings suggest that the Kiswahili translated and adapted version of the SF-12 could be used as an assessment tool for physical health, mental health and HSUV for Kiswahili-speaking PLHWA.

**Trial registration:**

Clinical trials.gov identifier: NCT00830622. Registered 26 January 2009.

## Background

Measures of health-related quality of life (HRQoL) and health state utility value (HSUV) measurements are vital components of healthcare program and technology evaluations. HRQoL is a multi-dimensional construct of an individual or group’s perceived health status, while HSUV ranks societal preferences for various states of health [1, 2]. HRQoL is used to measured functional changes in health as a clinical outcome of health interventions while HSUV describes the relative value that a society places on living in this health state. Although the two measures are related, they are theoretically distinct in their derivation, application and interpretation. Accurate measurement of HSUV and HRQoL require validated instruments. The limited number of validated instruments in East Africa has impeded studies evaluating the health or economic impact of new treatments or programs.

The 12-item Short-Form Health Survey (SF-12) is one of few instruments that can be used to evaluate both HRQoL and HSUV [3, 4]; however, it has not been validated for use in East Africa. The SF-12 measures eight dimensions of health to derive a physical component summary (PCS) and a mental health component summary (MCS). Further, an algorithm has been developed that converts SF-12 survey data into the preference-based Short Form 6D (SF-6D) score. The SF-6D provides information about the HSUV (based on the SF-12), which can be used to calculate quality-adjusted life years (QALY) [1]. The SF-12 and SF-6D are commonly used to collect HRQoL and HSUV for health technology evaluations in resource-rich settings, but they are rarely used in East Africa.

The SF-12 HRQoL scores provide descriptive measures of individual health, but not measures of economic value. The SF-12 PCS and MCS scores cannot be used directly to calculate quality-adjusted life years (QALY). Societal preferences of various health states are needed for cost-effectiveness evaluation of new health technologies, programs and interventions. Societal preferences of the general UK population have been elicited for a number of health states generated by the SF-12 using a time-trade off method [1]. An algorithm has been created based on these preferences to generate the SF-6D score, a value between 0.35 and 1. The SF-6D instrument scores are typically used directly in cost-effectiveness evaluations, and it is one of the most widely used intruments to estimate QALYs [1, 5].

HIV/AIDS is a progressive disease that results in a complex array of health states ranging from asymptomatic to severe opportunistic infection or HIV wasting syndrome. Additionally, antiretroviral therapy (ART) is associated with adverse events that can impact an individuals HRQoL. Kenya is an East African nation that has been seriously impacted by HIV with an estimated 1.5 million (1.3 million – 1.8 million) PLWHA in 2015 [6]. Because the SF-12 can describe a large range of health states, it can be a particularly useful tool to evaluate the health of people living with HIV/AIDS (PLWHA) at all stages of the disease. To date, the discriminative abilities of the PCS, MCS and SF-6D have not been investigated by HIV severity in East Africa. Given the two distinct purposes of these instruments (i.e., measuring health (SF-12) versus valuing health (SF-6D)), a validation of both instruments is needed regardless of the administration of the same questionnaire. Since HIV/AIDS is the leading cause of death in most East African nations [7], this validation is critically important for future use of the SF-12 to assess both health and economic outcomes. The objective of this study is to examine the performance of the Kiswahili-translated and adapted SF-12 survey, and the corresponding SF-6D scores, in Kenya. Particularly, the discriminative ability will be evaluated between well-defined severity groups in a sample of PLWHA.

## Methods

### Study design and setting

This cross-sectional study, which took place between May 2007 and October 2009, used data from a randomized controlled trial (RCT) in Nairobi, Kenya (*N* = 538) (ClinicalTrials.gov number, NCT00830622) [8]. Baseline data were collected prior to initiating ART or receiving the intervention. Data from participants in both trial arms were pooled to conduct these analyses. This multi-site trial involved three HIV clinics located in demographically and ethnographically diverse settings [8].

### Participants

Inclusion criteria were ART naïvety, aged 18 years or above, access to a mobile phone, and the ability to text message or have somebody who could text message on their behalf. Individuals who met the inclusion criteria and consented to participate were randomized to either receive a cell-phone based adherence intervention or standard care only. The study protocol was approved by the University of Manitoba and Kenyatta National Hospital ethics review boards [8]. The sample size calculation was based on primary trial outcomes including Viral load and adherence. While the trial was not specifically powered to measure the secondary HRQoL outcomes, a post-hoc sample size calculation revealed the sample was adequate to detect the MCID differences.

### Data and measures

The variables were defined at study entry, which took place at ART initiation. Individuals had been receiving care, but were ART naïve at the time of data collection. A translated and adapted SF-12 version one survey was administered to participants at baseline along with a survey that collected data on gender, age, income and rural/urban residence. The SF-12 was administered on the same day that the WHO stage, CD4 count and viral load measures were taken. CD4 count was collected (FACScan, Becton Dickinson, Sunnyvale, CA, USA) as part of routine clinical care and viral load (Amplicor, Roche Diagnostics, Mannheim, Germany) was assessed as part of the trial protocol [8]. Research clinicians administering the baseline survey assessed the World Health Organization (WHO) clinical stage of HIV infection [8].

### Theoretical foundation

A longer form of the SF-12, the SF-36, has been translated and adapted for use in 40 countries as part of the International Quality of Life Assessment (IQOLA) project [9]. Kiswahili, the primary language in many East African nations, was not among the original IQOLA project translations. However, two subsequent studies (Wagner et al. and Wyss et al.) translated and evaluated a Kiswahili translated SF-36 survey [10, 11]. Wagner et al. evaluated content, quality and scaling of the translated survey in a general Kenyan population, demonstrating that the SF-36 survey performed comparably to the UK counterpart [10]. Wyss et al. extended this work by assessing the validity of the SF-36 using a method of known group validation [11]. They demonstrated that the SF-36 could discriminate health status between groups with known differences in health based on theory or evidence. The discriminative ability of a HRQoL survey is an important validation step to ensure the survey can adequately capture outcomes of interest [12]. The SF-36 is cumbersome to administer in research settings, so the briefer SF-12 was created [3]. The SF-12 has been shown to retain much of the descriptive ability and validity of the SF-36, but has not been validated in East Africa.

### Translation and adaptation process

An international team of healthcare professionals and researchers translated the English SF-12 (Version 1) into Kiswahili based on IQOLA recommendations. The survey was reviewed by a multidisciplinary focus group of English and Kiswahili speaking healthcare providers and researchers for relevance, ease of understanding, and cultural appropriateness. Where necessary, items and response options were slightly modified and culturally adapted to make the questionnaire relevant and appropriate for use in a Kenyan context. Literature reviews and expert opinion were used to inform changes to the survey. For example, ‘climbing stairs’ in the original SF-12 was changed to ‘climbing a hill’, based on a previous study using the SF-36 in Tanzania [10, 11]. After translating the survey into Kiswahili, it was back translated into English and assessed by a focus group of English speaking healthcare researchers to ensure consistency. The survey was pre-tested on a sample of 20 Kenyan individuals and healthcare staff to evaluate cultural appropriateness and understanding.

### Validation

We investigated the construct validity of the survey using known group validation [11]. This method involves demonstrating that the PCS, MCS or SF-6D survey scores are able to discriminate scores between groups known a priori to have differences in their health status. We used three established criteria to classify HIV severity: CD4 cell count, viral load, and WHO clinical stage of HIV infection.

We hypothesized that the HRQoL and HSUV would be lower in more advanced HIV disease stages independently of how severity was defined. Further, since HIV is predominantly a physical disease, we hypothesized physical scores would show greater differences than mental health scores. Our specific hypotheses were: 1. MCS, PCS and SF-6D scores would be lower in individuals with CD4 < 200; MCS, PCS and SF-6D scores would be lower in individuals with viral load >55,000 copies/ml; and MCS, PCS and SF-6D scores would be lower in individuals in WHO stages 2, 3 & 4 compared to individuals in WHO stage 1. Since WHO stage 1 individuals are asymptomatic, we suspected that there would be a bigger difference in HRQoL and HSUV between these individuals and more symptomatic individuals [13].

### Severity threshold definitions

We used the United States (US) Center for Disease Control (CDC) severity stages, based on CD4 cell count, as our first definition of disease severity [14]. Stage 1 includes individuals with a CD4 count ≥500 cells/mm^3^; stage 2 includes individuals with a CD4 count between 200 and 499 cells/mm^3^; and stage 3 includes individuals with CD4 count <200 cells/mm^3^. The vast majority of individuals initiating ART have CD4 near or below 350 cells/mm^3^, as that was the ART treatment guidelines in Kenya at the time. Further, presentation to care with advanced HIV care has been defined as having a CD4 count below 200 [15]. To maintain an adequate sample in both groups, we dichotomized individuals above and below CD4 count of 200 cells/mm^3^, reflecting a comparison of individuals with advanced HIV infection to those without advanced HIV infection.

Our second definition of severity was based on a previous US study that used viral load threshold to classify individuals [12]. Viral load is associated with disease progression: an increased viral load indicates advanced disease and predicts progression to AIDS or death [16]. We classified individuals above or below 55,000 copies/ml to assess differences in the scores and draw descriptive comparisons to the previous US sample [12].

Our third definition of severity was the WHO HIV clinical staging system, which is based on physical symptoms. The WHO clinical stages are particularly useful in limited-resource settings, as CD4 cell counts are not always available. Symptoms have been grouped into four stages. Stage one individuals are asymptomatic; stage two individuals have mild symptoms such as rash or upper respiratory tract infections; stage 3 individuals have moderate to severe symptoms such as unexplained chronic diarrhea for greater than 1 month; and stage 4 individuals have severe to life-threatening symptoms such as extreme weight loss or opportunistic infections.

Based on our three definitions of severity, we categorized our sample into two groups based on their CD4 count or viral load threshold and four groups according to WHO clinical stages. We assessed the PCS, MCS and SF-6D, compared scores between each groups, and determined the discriminative ability of the scores.

### Statistical analysis

We conducted a descriptive analysis of the baseline characteristics of the study population, and stratified the results by the severity groups we defined. We calculated individual PCS and MCS scores using correlated weights from the US and SF-6D scores based on UK weights [1, 3, 17, 18]. The SF-12 was designed to give a population mean MCS and PCS of 50 with a standard deviation of 10 in a disease-free US population [3]. The minimum clinically significant difference (MCID) for both PCS and MCS scores has been suggested to be in the range 3–5 points; however, MCID for HRQoL scores are not well-established [19]. We used a change of 3 to interpret the clinical significance of differences that we observed, but caution is suggested in interpreting the MCID since a 1-point change can be meaningful if it came at no additional cost [19]. The MCID for the SF6D has been suggested to be 0.033 (95% CI 0.029 to 0.037) [20].

We calculated mean PCS, MCS and SF-6D scores in each of the severity categories. For CD4 and viral load threshold analyses, t-tests were used to test for statistical differences between the two groups. For the WHO clinical stage analysis, we used analysis of variance analysis (ANOVA) with a post-hoc analysis to test for differences in scores between the four groups. Participants with missing CD4 counts, viral load or WHO stage were excluded from the respective analysis.

We used receiver operator characteristic (ROC) curves as a second test of the discriminative ability of the instruments [12, 21]. Traditionally, a ROC plots the sensitivity by 1-specificity of a diagnostic test and helps to determine the ability of the test to discriminate between a diseased and non-diseased population. It has also previously been used to determine the construct validity of an instrument by evaluating if the instrument can correctly discriminate two groups known to have differing HRQOL [12]. We used ROC curves to assess whether the scores could correctly categorize a participant into a severity group using different threshold scores as cut-offs. The area under the ROC curve (AUC) is a measure of signal to noise of an instrument [21]. An AUC of 1 indicates perfect discriminatory ability; an AUC of between 0.8 to 1 shows good to excellent ability to discriminate; an AUC of between 0.7 to 0.8 shows fair discriminative ability; an AUC of between 0.60 and 0.70 shows weak ability to discriminate; an AUC below 0.60 indicates a failure to discriminate between groups; and an AUC of 0.50 suggests the instrument is no more useful to predict the group to which an individual belongs than flipping a coin [21].

## Results

The sample had 538 participants, with greater representation by females (*n* = 350/538; 65%) and urban residents (*n* = 436/538, 81%). Table 1 shows the characteristics of our sample separated by severity category. CD4 count data were complete; however, 9 (1.7%) participants had missing SF-12 responses; 43 (8.0%) were missing viral load data; and 72 (13.3%) were missing WHO clinical stage. Table 2 summarizes the mean scores by severity group and Table 3 lists the AUC results of each score. We observed statistically and clinically significant differences in PCS scores in several comparisons. The MCS had a weak signal in some comparisons, indicating that had a modest ability to discriminate across groups. The SF-6D scores also show monotonic trends in the hypothesized direction in all analyses and there were statistically significant differences in several comparisons (Table 2).

**Table 1:** Characteristics of sample separated by severity category

	CD4 < 200 *N* = 364 *N* (%)	CD4 ≥ 200 *N* = 169 *N* (%)	VL^a^ >55,000 *N* = 281 *N* (%)	VL^a^ ≤55,000 *N* = 214 *N* (%)	Stage 1 *N* = 114 *N* (%)	Stage 2 *N* = 126 *N* (%)	Stage 3 *N* = 204 *N* (%)	Stage 4 *N* = 22 *N* (%)
Male Gender	136 (37)	51 (30)	114 (41)	62 (29)	30 (26)	48 (38)	72 (35)	7 (32)
Age								
20–29	62 (17)	37 (22)	46 (16)	43 (20)	30 (26)	19 (15)	39 (19)	4(18)
30–39	184 (51)	88 (52)	148 (53)	104 (49)	63 (55)	59 (47)	95 (47)	10 (45)
40–49	89 (24)	32 (19)	68 (24)	48 (22)	16 (14)	24 (19)	51 (25)	8 (36)
50+	30 (8)	12 (7)	19 (7)	19 (9)	5 (4)	3 (2)	19 (9)	0 (0)
Income (Schillings)								
≤ 2000	93 (29)	43 (29)	58 (23)	65 (35)	26 (27)	29 (25)	57 (32)	6 (30)
2001–10,000	140 (43)	71 (48)	114 (45)	80 (43)	41 (43)	59 (51)	84 (48)	4 (20)
10,001–40,000	75 (23)	30 (20)	64 (25)	36 (19)	25 (26)	24 (20)	27 (15)	10 (50)
> 40,000	14 (4)	5 (3)	15 (6)	4 (2)	3 (3)	3 (2)	8 (5)	0 (0)
Urban Res.	295 (81)	139 (82)	238 (85)	170 (79)	107 (94)	116 (92)	137 (67)	18 (82)

^a^ *VL* viral load

**Table 2:** Mean HRQoL scores by severity subgroup

Sub Group	PCS (SD^a^)	MCS (SD^a^)	SF6D (SD^a^)
CD4 < 200 *N* = 364	41.1 (11.0)^*^	43.4 (10.7)^*^	0.67(0.15)^*^
CD4 ≥ 200 *N* = 169	45.4 (10.3)^*^	45.8 (11.0)^*^	0.72(0.15)^*^
Viral Load >55,000 *N* = 281	41.5 (10.6)^*^	43.8 (10.9)	0.67 (0.15)^*^
Viral Load ≤55,000 *N* = 214	43.7 (11.3)^*^	44.5 (10.8)	0.71 (0.16)^*^
WHO Stage 1 *N* = 114	46.7 (8.7)^**^	46.0 (11.0)^**^	0.73 (0.15)^**^
WHO Stage 2 *N* = 126	44.7 (10.3)	44.6 (10.3)	0.71 (0.15)
WHO Stage 3 *N* = 204	39.5 (11.3)^**^	42.7 (11.0)	0.66 (0.16)^**^
WHO Stage 4 *N* = 22	36.9 (11.3)^**^	41.6 (10.2)^**^	0.61 (0.13)^**^

^a^Standard Deviation

*Statistically significant difference between severity group *p* < 0.05

**Statistically significant difference between severity group *p* < 0.05 based on ANOVA with post-hoc Tukey’s procedure

**Table 3:** Area under the ROC curve comparisons

Comparison Groups	PCS AUC	MCS AUC	SF6D AUC
CD4 < 200 vs CD4 ≥ 200	0.61	0.61	0.61
Viral Load ≤55,000 vs >55,000	0.56	0.54	0.57
WHO stage 1 vs stage 2	0.55	0.58	0.55
WHO stage 1 vs stage 3	0.67	0.67	0.64
WHO stage 1 vs stage 4	0.72	0.71	0.68

### Results by CD4 count threshold severity definition

**Fig. 1:**
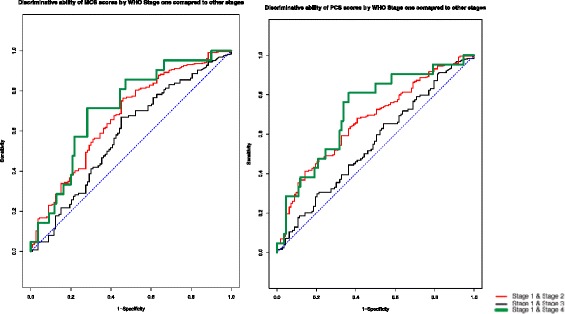
The PCS and SF-6D ROC curves when comparing WHO stage one to more advanced stages. Caption: The area under the ROC curve (AUC) is a measure of signal to noise of an instrument. The signal appears to improve as the severity gap between the comparison groups increases. This indicates discriminatory ability of both survey scores and gives face validity to them since the survey is correctly measuring what it was designed to measure

**Fig. 2:**
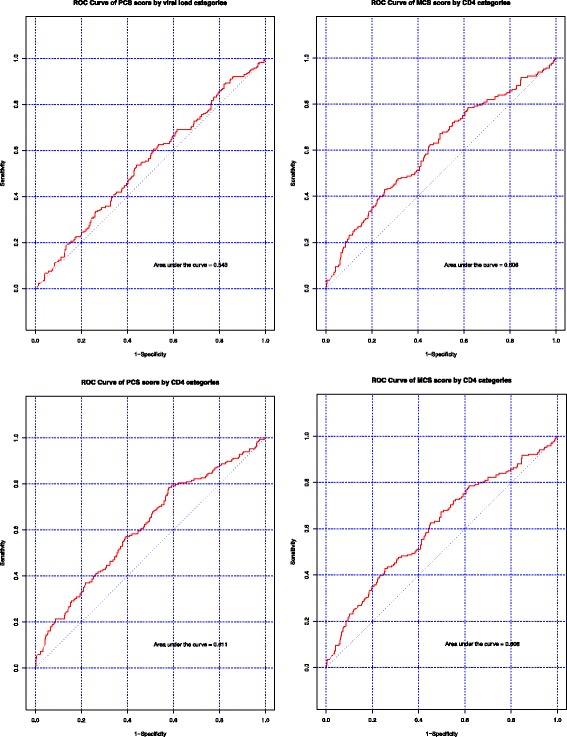
The ROC curves of SF-12 derived PCS and MCS using CD4 and viral load thresholds. Caption: The signal was weaker in this comparison, partly because of the more general definitions of severity. However, both PCS and MCS showed some signal by CD4 severity threshold comparison

**Fig. 3:**
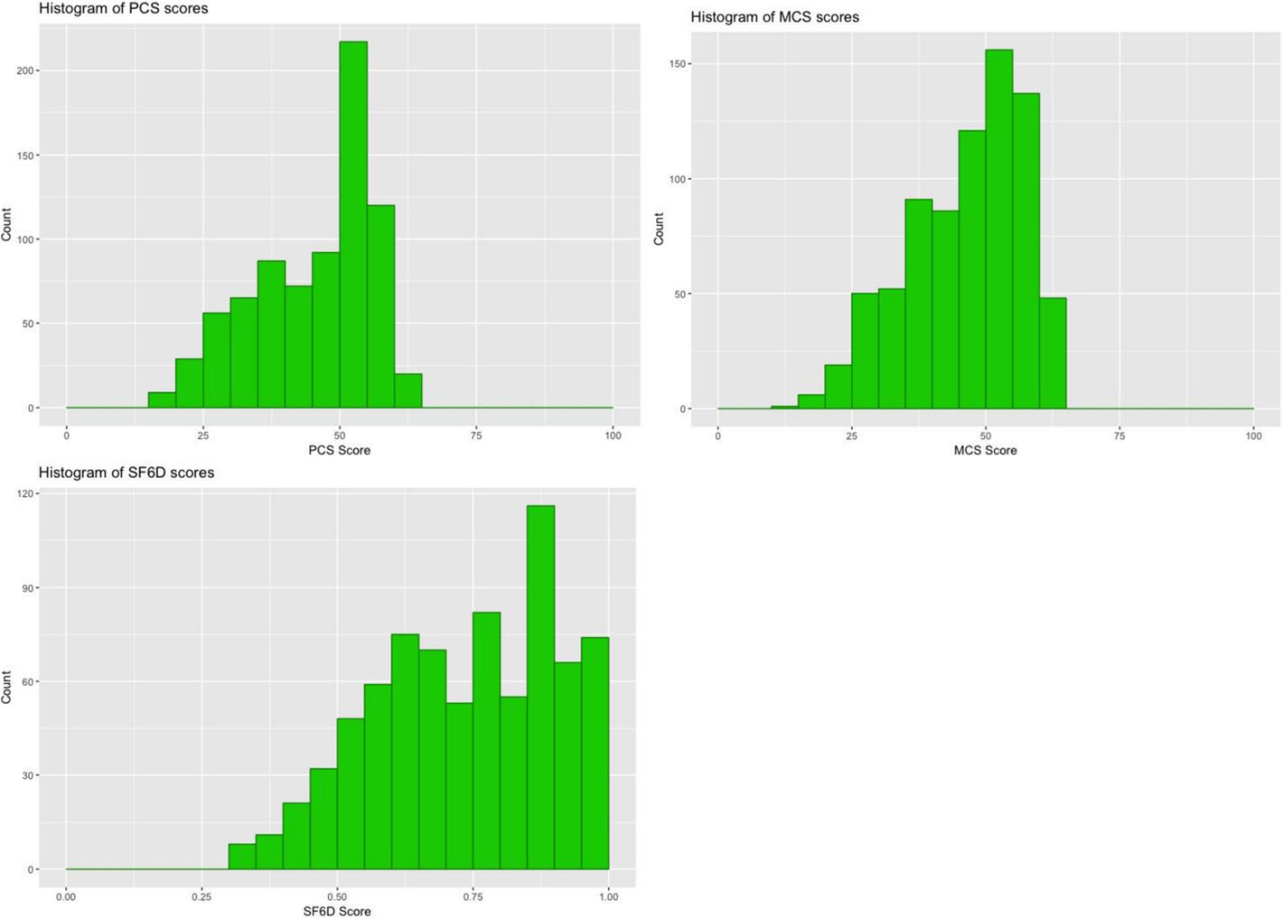
Histogram of survey scores. Caption: The PCS and MCS scores did not appear to have any floor or ceiling effects in this sample. However, the SF6D may have had both a floor effect at a score of 0.3 and a ceiling effect at a score of 1

Mean PCS and SF-6D scores were significantly lower in individuals with CD4 < 200 cells/mm^3^ than in individuals above that threshold. The PCS was 4.3 units lower and the SF6D was 0.05 units lower suggesting a clinically significant difference based on the MCID. The mean MCS score was 2.4 units lower in individuals with CD4 < 200 cells/mm^3^, so the difference was not clinically significant. We also compared mean values of PCS and MCS scores to a US sample and scores from of our sample were comparable to the previously reported estimates (Table 4) [12]. The AUC for all three scores were in the weak to poor range (Figs. 1 and 2), indicating that they had some ability to distinguish these severity groups (0.57–0.61). Floor and ceiling effects were observed for the SF6D scores, but not for the PCS and MCS scores (Fig. 3).

**Table 4:** Comparison of mean scores to a US sample of HIV patients

	PCS Kenya Mean (SD^a^)	MCS Kenya Mean (SD)	PCS USA [11] Mean (SD)	MCS USA [11] Mean (SD)
CD4 ≥ 200 cells/mm^3^	45.4 (10.3)	45.8 (11.0)	45.3(11.3)	42.6 (9.6)
CD4 < 200 cells/mm^3^	41.1 (11.0)	43.4 (10.7)	40.1 (11.4)	43.3(9.8)
Viral load ≤55,000 copies/ml	43.7 (11.3)	44.5 (10.8)	44.5 (11.6)	42.9 (9.5)
Viral load >55,000 copies/ml	41.5 (10.6)	43.8 (10.9)	40.2 (11.5)	41.6 (10.2)

^a^Standard Deviation

### Results by viral load threshold

The SF-6D score was statistically significantly lower in individuals with a viral load >55,000 copies/ml and met the MCID difference we specified. The PCS and MCS scores were also lower, but were not clinically significant according to the MCID we specified. The average scores within these viral load categories were comparable to a previously reported US sample (Table 4). The AUC was poor, indicating that the survey could not discriminate well between these populations (Figs. 1 and 2).

### Results by WHO stage

Both the PCS and SF-6D had a statistically significant monotonic downwards trend as severity increased. The difference in PCS scores between stage 1 and stages 2, 3 and 4 was 2.0, 7.2 and 9.8 units respectively, indicating a clinically significant difference in physical health as HIV progresses from stage 1 through 4. The AUC of the PCS and SF-6D were 0.71 and 0.68, respectively, indicating that the scores had fair discriminate ability between WHO stages one and four (Fig. 1). The MCS means were statistically different between Stage 1 and Stage 4 patients and the AUC by this comparison was 0.71 suggesting ability to discriminate between these two groups.

## Discussion

Our study shows that HRQoL and HSUV scores derived from a Kenyan modified and translated SF-12 survey can discriminate HIV disease severity using three severity definitions. These findings suggest construct validity of the modified SF-12 and may have important implications for the use of the instrument in Kenya and other east African nations. We confirm that the SF-12 may be used as a tool to measure physical and mental health as part of program and intervention evaluations. Furthermore, the SF-12 survey can be scored to derive an SF-6D preference-based measure that can be used to calculate QALYs. The SF-6D scores declined with increased severity of disease and could theoretically rank health states in a valid order in practice. These instruments could be particularly important to support the increasing demand for measurement and evaluation of HIV/AIDS programs. Additionally, our results have described the mean and distribution of HRQoL or HSUV scores for a variety of HIV health states, and the results could be used in mathematical models to calculate QALYs, estimate disease burden and/or conduct economic evaluations in Kenya.

The WHO stages were perhaps the most accurate indication of HRQoL since the system relies on the present or absence of a variety of symptoms based on HIV severity. Data were collected by highly trained research nurses as part of an internationally funded randomized trial adding a level of scrutiny to data collection and accuracy of classification. As would be expected, we observed the largest differences in PCS, MCS and SF-6D between WHO stage 1 and stage 4. We were unable to find a similar comparison in the literature, but the findings confirm the survey’s ability to discriminate between groups known to have differences in HRQoL. We also observed differences in groups by established CD4 and viral load thresholds. We were able to confirm the survey scores could discriminate between these alternate classifications as further confirmation of the discriminatory ability of the survey scores. Our findings were strengthened through the consistent findings across multiple criteria of HIV severity.

Our results were consistent with previous studies of HRQoL and HSUV in PLWHA. Delate et al. reported mean SF-12 summary scores in a sample of US PLWHA [12]. The mean PCS and MCS scores we observed in a Kenyan population have mostly similar means and standard deviations as the US sample. In a systematic review of HIV/AIDS focused HSUV studies, Tengs et al. pooled utility values for three HIV health states: asymptomatic HIV; symptomatic HIV; and AIDS; they reported HSUVs of 0.94, 0.82 and 0.70 respectively [13]. The mean HSUVs in our sample were generally lower (0.61–0.73) than those reported in the systematic review (Table 2). However, the review summarized evidence of HSUV of a broad sample of PLWHA, while we assessed a cohort at a particularly vulnerable time: ART initiation. Within severity groups, the average HSUV may have improved over time due to adaptation to disease and due to drug treatment [22].

There were several limitations to this study. First, normative data from the United States was used to calculate the PCS and MCS and scoring data from the UK was used to calculate the SF6D scores. External scoring was used due to a lack of a local scoring algorithm for the SF-12 or SF6D in Kenya or a similar setting. Previous studies in Africa have used scoring data from other settings as a surrogate to overcome this limitation, however future studies are needed to evaluate these important measures in Kenya and other African settings [23, 24]. Second, we were missing WHO stage and viral load data for several participants. We had an adequate sample size to show statistically significant differences between groups; however, the direction of the potential bias due to missing data is uncertain. Since the missing data may have been due to administrative errors, there would likely be no systematic pattern in missing individuals. Finally, the survey had been modified from its original questions, so theoretical constructs may have been affected. The survey appears to perform as designed in main scores derived from the survey, but more nuanced measures of health status were not assessed in this study.

## Conclusion

We found that a Kiswahili translated and adapted SF-12 survey could discriminate between HIV severity groups in Kenya. The SF-12 is widely used in clinical trials in the US and Europe as an objective measure of HRQoL associated with new drug therapies and health interventions. The SF-12 could accompany clinical trials being conducted in Kenya and in other areas in East Africa to help quantify HRQoL and HSUV that have previously gone unmeasured. Further research is needed to show the ability of the SF-12 survey to detect changes in quality of life over time as individuals’ health status changes. Further research is also needed to determine Kenya specific scoring for both the SF-12 and SF-6D instruments, and to test the survey in a broad range of diseases. This study is a fundamental step towards increased use of the SF-12 and other HRQoL instruments in east Africa.

## Abbreviations

AIDS: Acquired immunodeficiency syndrome
ART: Antiretroviral therapy
AUC: Area under the curve
HIV: Human immunodeficiency virus
HRQoL: Health related quality of life
MCID: Minimum clinically important difference
MCS: Mental component summary
PCS: Physical component summary
ROC: Receiver operating characteristic
SF 12: Short form 12 questionnaire
SF6D: Short form 6 dimension score
US: United States
WHO: World Health Organization
